# Effectiveness of the continuing care model for improving quality of life, cardiac function and outcomes in patients with coronary heart disease combined with heart failure after percutaneous coronary intervention: A retrospective study

**DOI:** 10.12669/pjms.41.10.12538

**Published:** 2025-10

**Authors:** Chunyan Huang, Dan Liu, Jingjing Lin

**Affiliations:** 1 Chunyan Huang, Department of Cardiovascular Medicine, Affiliated Jinhua Hospital, Zhejiang University School of Medicine, Jinhua, Zhejiang Province 321000, P.R. China; 2 Dan Liu, Department of Cardiovascular Medicine, Affiliated Jinhua Hospital, Zhejiang University School of Medicine, Jinhua, Zhejiang Province 321000, P.R. China; 3 Jingjing Lin, Department of Cardiovascular Medicine, Affiliated Jinhua Hospital, Zhejiang University School of Medicine, Jinhua, Zhejiang Province 321000, P.R. China

**Keywords:** Continuing care, Coronary heart disease combined with heart failure, Percutaneous coronary intervention, Quality of life, Cardiac function, Ventricular remodeling, Major adverse cardiovascular events

## Abstract

**Background & Objective:**

Percutaneous coronary intervention (PCI), the mainstay for coronary heart disease (CHD) treatment, is associated with some adverse cardiovascular events (ACEs). This study aimed to assess the impact of different nursing care models on clinical outcomes in patients with coronary heart disease (CHD) complicated by heart failure (HF) following percutaneous coronary intervention (PCI).

**Methodology:**

This was a single-center retrospective observational study conducted at the Department of Cardiovascular Medicine, Affiliated Jinhua Hospital, Zhejiang University School of Medicine, involving 183 patients diagnosed with CHD combined with HF who underwent PCI between May 2021 and May 2023. Patients were categorized into a continuing care group or a routine care group according to the post-PCI care model they had received. Quality of life was assessed using the Health-Promoting Lifestyle Profile II (HPLP-II) and the Short Form-36 (SF-36). Cardiac function was evaluated through the six minutes’ walk test (6-MWT), left ventricular ejection fraction (LVEF), and N-terminal pro-B-type natriuretic peptide (NT-proBNP) levels. All participants were followed up for six months after PCI to record ventricular remodeling and major adverse cardiovascular events (MACEs).

**Results:**

After six months of follow-up, the continuing care group showed significantly higher HPLP-II and SF-36 scores, LVEF, and 6-MWT results, and lower NT-proBNP levels than the routine care group. Compared with routine care, continuing care was associated with a significantly lower incidence of ventricular remodeling (17.20% vs. 30.00%) and MACEs (44.09% vs. 66.67%).

**Conclusion:**

Continuing care has the potential to enhance quality of life and cardiac function in patients with CHD and HF following PCI while reducing the incidence of ventricular remodeling and MACEs.

***Note:*** A continuing care team was established to assess the health status of CHD patients with heart failure after PCI and propose corresponding intervention strategies.

## INTRODUCTION

Coronary heart disease (CHD) is a cardiovascular disease defined as a composite of coronary death, coronary revascularization, or myocardial infarction^1^,^2^ and remains the predominant cause of morbidity and mortality worldwide.^3^ Additionally, CHD patients have a high lifetime risk of developing heart failure (HF).^4^,^5^ Although percutaneous coronary intervention (PCI) has long been the mainstay of CHD treatment, it is still associated with adverse cardiovascular events (ACEs) in patients despite current developments in postoperative secondary prevention therapies.^6^,^7^ Therefore, more effective secondary prevention strategies are needed to minimize ACE following PCI.

Continuing supportive care is considered an effective disease management model for patients with CHD and has evolved significantly over time.^8^-^12^ In recent years, the integration of mobile health (mHealth) platforms, remote symptom monitoring, and digital adherence tools has extended the reach of continuing care beyond traditional in-person interactions.^13^,^14^ These innovations enhance communication, support early detection of deterioration, and empower patients in self-management, particularly in post-discharge cardiovascular care. Studies have showed that the model is efficacious in improving cardiac and pulmonary function, medication compliance, and quality of life has been proven in lung cancer patients with CHD undergoing PCI,^15^ can improve the cardiac function of CHD patients after PCI and increase their quality of life and medication compliance, eventually facilitating cardiac rehabilitation.^16^ Additionally, the implementation of the continuing care model in patients with chronic HF was associated with improved self-care behaviors, one of the most predominant determinant factors for the prognosis of HF.^17^ According to recent data, mutual goal-based continuing care confers a beneficial effect on the outcomes of CHD patients undergoing PCI, enhancing self-management, quality of life, and satisfaction of patients and reducing readmission rates.^18^ However, few reports have focused on the effect of continuing care on adverse events in post-PCI patients with CHD combined with HF.

This study aimed to investigate the impact of continuing care on health outcomes, such as quality of life, cardiac function, ventricular remodeling incidence, and major adverse cardiovascular events (MACEs), in CHD patients with HF after PCI.

## METHODOLOGY

This was a retrospective, single-center observational study conducted at the Department of Cardiovascular Medicine, Affiliated Jinhua Hospital, Zhejiang University School of Medicine. A total of 183 patients with CHD and HF who underwent PCI between May 2021 and May 2023 were included in this analysis.

### Inclusion criteria:

- Meeting the diagnostic criteria for CHD combined with HF.^19^,^20^
- Eligible for PCI surgery.
- Age between 18 and 70 years.
- Receiving optimal medical therapy (OMT), including aspirin, clopidogrel, statins, beta-blockers, angiotensin-converting enzyme inhibitors, or angiotensin II receptor blockers.
- Good mental condition and communication ability.

### Exclusion criteria:

- New York Heart Association (NYHA) functional Class-III or IV.
- Allergy to any OMT medications.
- Combined severe organ damage.
- Severe cardiogenic shock.
- Severe liver and kidney failure.
- Severe bleeding tendency (alanine aminotransferase/aspartate aminotransferase < 40 μmol/L, serum creatinine > 1330 μmol/L).
- HF caused by cardiomyopathy, rheumatic heart disease, severe valvular heart disease, hypertension, and chronic obstructive pulmonary disease.
- Life expectancy of less than one year.
- Breastfeeding or pregnant women;
- Significant missing clinical data.
- Loss to follow-up.

### Exclusion criteria:

One hundred eighty-three patients were included in this study. This study collected demographic and clinical information of participants, including age, gender, body mass index (BMI), history of hypertension, diabetes, atrial fibrillation, smoking history, drinking history, education level, place of residence, marital status, family income, NYHA functional class, and PCI type.

### Ethical Approval:

The study was reviewed and approved by the Medical Ethics Committee of the Affiliated Jinhua Hospital, Zhejiang University School of Medicine (Approval No. 2021-109; dated October 10, 2025). All procedures involving human participants were conducted in accordance with the ethical standards of the institutional and/or national research committee and with the 1964 Helsinki Declaration and its later amendments. Written informed consent was obtained from all participants prior to inclusion.

CHD was confirmed by coronary angiography or coronary CT showing at least 50% stenosis in any of the major vessels (left main coronary artery, left anterior descending artery, left circumflex artery, or right coronary artery). Patients presented with symptoms of NYHA functional classes II-IV. Class-II patients with heart disease presented with mild limitation in physical activity, no conscious symptoms at rest, and excessive fatigue, palpitations, asthma, or angina after general physical activities. Class-III patients with heart disease presented with significantly limited physical activity, no symptoms at rest, and excessive fatigue, palpitations, asthma, or angina after less-than-general physical activity. Class-IV patients with heart disease could engage in any physical activity and experienced HF symptoms even at rest, which were aggravated after physical activity. All patients with CHD combined with HF included in this study belonged to NYHA functional Class-II.^20^,^21^

Blood biomarkers were collected and measured both at admission and six months after the procedure. Serum levels of uric acid (UA), creatinine (Cr), total cholesterol (TC), triglyceride (TG), high-density lipoprotein cholesterol (HDL-C), low-density lipoprotein cholesterol (LDL-C), cystatin C (CysC), hypersensitive C-reactive protein (hs-CRP), and N-terminal pro B-type natriuretic peptide (NT-proBNP) were measured using corresponding ELISA kits (Gelatin, China; JLC-G5037; JLC-G4647; JLC-R13720; JLC-G4417; JLC19997; JLC-G5075; JLC-G3746; JLC-A9005; JLC-G4549, respectively). All procedures strictly adhered to the manufacturer’s guidelines. The left ventricular end-diastolic volume (LVEDV) and left ventricular end-systolic volume (LVESV) of all participants were determined by Doppler echocardiography (DC-38, Mindray, China). LVEF was calculated as LVEF = (LVEDV - LVESV)/LVEDV × 100%.

Cardiopulmonary function was assessed by the six minutes’ walk test (6-MWT), a commonly used clinical method to evaluate cardiopulmonary function by measuring the distance an individual can walk quickly on a flat, hard surface within six minutes. This test evaluates the comprehensive response of all body systems during exercise, including the lungs, cardiovascular system, systemic circulation, blood, neuromuscular units, and muscle metabolism.^22^

The extent to which adults engage in a health-promoting lifestyle was measured using the Health-Promoting Lifestyle Profile-II (HPLP-II), which comprises 48 items covering six domains: physical activity, interpersonal support, nutrition, health responsibility, self-actualization, and stress management. A Likert scale of 4 points was used: never = 1 point, occasionally = 2 points, often = 3 points, and always = 4 points. The total score ranges from 48 to 192 points, with higher scores indicating better outcomes.^23^

The Short Form 36 (SF-36) was used to assess the quality of life. SF-6 is divided into eight dimensions: physical functioning, role physical, bodily pain, general health perceptions, social functioning, role emotional, and mental health. The total score of the scale is 100, with higher scores reflecting better quality of life.^24^ While data for all subscales were collected, only the total SF-36 score was analyzed in this study to provide a simplified global indicator of health-related quality of life. This approach was adopted to maintain statistical clarity and avoid over-fragmentation of the results.

### Care programs:

*Routine care:* Patients in the routine care group received standard postoperative care during their hospital stay. This included a brief introduction to the environment, such as explaining the “Help” button in the ward. Additionally, patients received disease and health education, diet and exercise guidance, psychological counseling and care, medication guidance, and post-discharge follow-up. Patients were also informed to undergo a review six months after the surgery.

### Continuing care:

A continuing care team was established to assess the health status of CHD patients with heart failure after PCI and propose corresponding intervention strategies. Additionally, the care team provided professional skills to patients, their families, and the community to help reduce or prevent related symptoms. For example, patients were informed on how to use antiplatelet drugs to monitor for subcutaneous bleeding, how to use statins to monitor and to avoid liver damage and myopathy, and how to handle emergencies like recurrent angina.

Beyond general health education, PCI-related information, brochures, operational procedures, and videos were provided to patients, families, and the community to enhance health education.

Patients were monitored and tracked monthly in various ways, effectively improving medication compliance. Using social network tools to manage patients after PCI increased their awareness of self-management and improved the management provided by families and the community. Additionally, the care team helped answer questions from families and organizations. Continuing care lasted six months, with a review conducted six months after PCI.

### Follow-up:

All patients with CHD combined with HF were followed up six months after PCI to record ventricular remodeling and MACEs. Ventricular remodeling was diagnosed per a ≥ 20% increase in LVEDV from baseline 6 months after PCI.^25^ MACEs included unstable angina, stroke, myocardial infarction, late revascularization, and all-cause mortality.^26^,^27^ Definitions for each event were based on the 2020 ESC guidelines. Specifically, unstable angina was defined as new or worsening chest pain at rest or minimal exertion, accompanied by ischemic ECG changes without elevation of cardiac biomarkers, requiring hospitalization or urgent evaluation. Late revascularization was defined as repeat PCI or CABG occurring >30 days after the index procedure, performed due to recurrent ischemic symptoms or objective ischemia confirmed by non-invasive imaging. All events were reviewed and adjudicated independently by two attending cardiologists who were blinded to group assignments.

### Statistical analysis:

Data analysis and graphing were performed using SPSS 21.0 software, MedCalc software (version 22.2), and GraphPad Prism 8.0.1 software. Data examined by the Kolmogorov-Smirnov test were consistent with a normal distribution, and measurement data were presented as mean ± standard deviation. The independent sample t-test was used to compare the differences between the two groups. Pre- and post-care data were compared using the paired t-test. Data with a non-normal distribution were presented as median (minimum-maximum), and the Mann-Whitney U test was employed to compare differences between the two groups. Count data were presented as numbers and percentages, and the chi-square test was used for intergroup comparison. A two-tailed *p*-value < 0.05 indicated statistical significance, with α = 0.05 set as the inspection level.

## RESULTS

**Fig.1:**
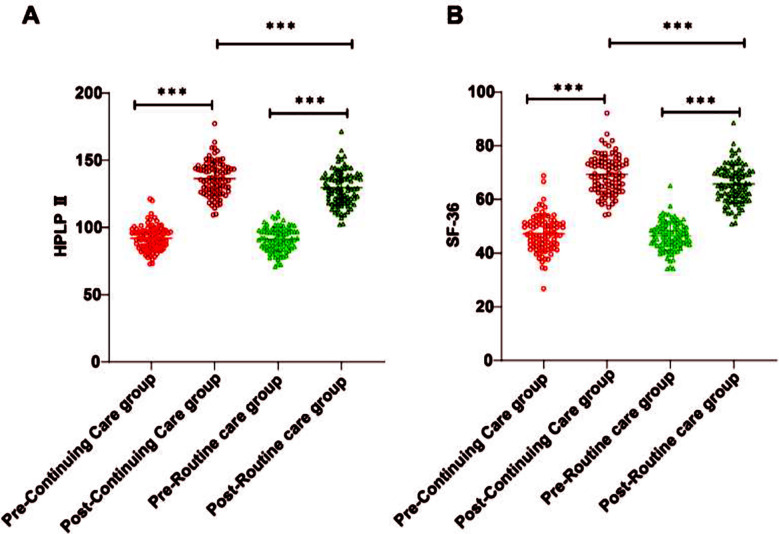
Comparison of quality of life between the continuing and routine care groups. A, HPLP-II scores of patients in the continuing and routine care groups before and after care. B, SF-36 scores of patients in the continuing and routine care groups before and after care. ***, *p* < 0.001.

No significant differences between the two groups in baseline characteristics (all *p* > 0.05), Table-I. HPLP-II and SF-36 were used to assess the quality of life of CHD patients with HF before and after care. As shown in Fig.1, HPLP-II and SF-36 scores were comparable in both groups before care (*p* > 0.05). However, after care, both groups showed an upward trend in HPLP-II and SF-36 scores (*p* < 0.001), with significantly higher scores in the continuing care group compared to the routine care group (*p* < 0.001).

**Table-I:** Comparison of baseline data of patients between Continuing and Routine care groups.

Items	Continuing care group (n =93)	Routine care group (n=90)	z/t/x^2^	p
Age (year)	54 (42, 66)	54 (40, 68)	0.424	0.671
Gender (male/female)	63/30	57/33	0.394	0.530
BMI (kg/m^2^)	23.36 (14.77, 32.12)	23.76 (18.34, 29.06)	0.927	0.354
History of hypertension (case, %)			1.637	0.201
Yes	19 (20.43)	12 (13.33)		
No	74 (79.57)	78 (86.67)		
History of diabetes (case, %)			1.330	0.249
Yes	31 (33.33)	23 (25.56)		
No	62 (66.67)	67 (74.44)		
History of atrial fibrillation (case, %)			1.977	0.160
Yes	42 (45.16)	50 (55.56)		
No	51 (54.84)	40 (44.44)		
Smoking history (case, %)			2.047	0.153
Yes	24 (25.81)	32 (35.56)		
No	69 (74.19)	58 (64.44)		
Drinking history (case, %)			1.588	0.208
Yes	26 (27.96)	33 (36.67)		
No	67 (72.04)	57 (63.33)		
Education (case, %)			1.027	0.311
Elementary school, junior high school	51 (54.84)	56 (62.22)		
High school and above	42 (45.16)	34 (37.78)		
Marital status (case, %)			0.665	0.415
Married	62 (66.67)	65 (72.22)		
Single, divorced, or widowed	31 (33.33)	25 (27.78)		
Place of residence (case, %)			3.764	0.052
Township	50 (53.76)	61 (67.78)		
City	43 (46.24)	29 (32.22)		
Family income (month, case, %)			0.182	0.670
≤ 5000	38 (40.86)	34 (37.78)		
> 5000	55 (59.14)	56 (62.22)		
PCI type (case, %)			1.322	0.724
Balloon angioplasty	28 (30.11)	23 (25.56)		
Stent thrombosis	56 (60.22)	61 (67.78)		
Thrombus aspiration	2 (2.15)	1 (1.11)		
Other	7 (7.53)	5 (5.56)		
UA (μmol/L)	462.79 ± 54.47	457.191 ± 53.33	0.703	0.483
Cr (μmol/L)	75.00 ± 6.89	75.33 ± 6.92	0.323	0.747
TC (mmol/L)	4.73 ± 1.14	4.42 ± 1.45	1.627	0.105
TG (mmol/L)	1.81 ± 0.55	1.77 ± 0.50	0.657	0.512
HDL-C (mmol/L)	1.20 ± 0.33	1.21 ± 0.31	0.194	0.847
LDL-C (mmol/L)	2.60 ± 1.00	2.52 ± 0.89	0.513	0.608
CysC (mg/L)	1.60 ± 0.61	1.59 ± 0.59	0.155	0.877
hs-CRP (mg/L)	3.63 ± 0.96	3.68 ± 0.99	0.346	0.730

***Note:*** BMI, body mass index; PCI, percutaneous coronary intervention; UA, uric acid; Cr, creatinine; TC, total cholesterol; TG, triglyceride; HDL-C, high-density lipoprotein cholesterol; LDL-C, low-density lipoprotein cholesterol; CysC, Cystatin C; hs-CRP, hypersensitive C-reactive protein; NT-proBNP, N-terminal pro B-type natriuretic peptide.

### Cardiac function assessment:

**Fig.2:**
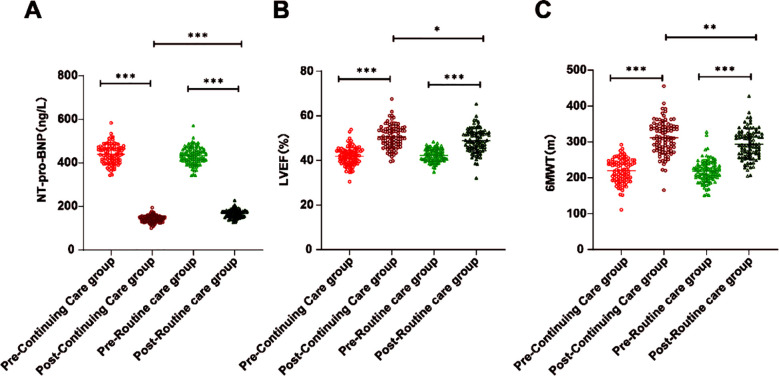
Comparison of cardiac function between the continuing and routine care groups before and after care. A, Comparison of serum NT-proBNP levels between the continuing and routine care groups before and after care. B, Comparison of LVEF between the continuing and routine care groups before and after care. C, Comparison of 6-MWT between the continuing and routine care groups before and after care. * *p* < 0.05, ** *p* < 0.01, *** *p* < 0.001.

There were no significant differences in the NT-proBNP, LVEF, as well as the 6-MWT score between the groups before care (all *p* > 0.05), Fig.2. After care, LVEF and 6-MWT increased, and NT-proBNP decreased in both groups (all *p* < 0.001). Post-care LVEF and 6-MWT were significantly higher in the continuing care group, and the NT-proBNP level was considerably lower than in the routine care group (all *p* < 0.05).

### Assessment of ventricular remodeling:

The incidence of ventricular remodeling in the continuing care and routine care groups were 17.20% (16/93) and 30.00% (27/90), respectively. Additionally, the incidence rate of ventricular remodeling in the continuing care group after care was significantly lower than in the routine care group (*p* < 0.05), Table-II.

**Table-II:** Comparison of the incidence of ventricular remodeling between continuing and routine care groups.

	ventricular remodeling	no ventricular remodeling
Continuing care group (n = 93)	16 (17.20)	77 (82.80)
Routine care group (n = 90)	27 (30.00)	63 (70.00)
*x* ^2^	4.166	
*p*	0.041	

***Note:*** Count data were displayed as the number of cases and percentages, which were compared between groups using the chi-square test.

### Assessment of MACEs:

The incidence of MACEs were 44.09% (41/93) in the continuing care group and 66.67% (60/90) in the routine care group. The incidence of MACEs was significantly lower in the continuing care group compared to the routine group (*p* < 0.01), Table-III.

**Table-III:** Comparison of the incidence of MACEs between continuing care and routine care groups.

	Unstable angina	Stroke	Myocardial infarction	Late revascularization	All-cause mortality	Total incidence rates of MACE
Continuing care group (n = 93)	14 (15.05)	6 (6.45)	8 (8.60)	10 (10.75)	3 (3.23)	41 (44.09)
Routine care group (n = 90)	18 (20.00)	7 (7.78)	14 (15.56)	17 (18.89)	4 (4.44)	60 (66.67)
*x* ^2^						4.241
*p*						0.009

***Note:*** Count data were displayed as the number of cases and percentages, which were compared between groups using the chi-square test.

## DISCUSSION

The current study revealed that implementing continuing care for post-PCI patients with CHD complicated by HF could effectively improve their quality of life and cardiac function and reduce the rate of adverse events, such as ventricular remodeling and MACEs. This care approach may provide further insights into treatment response and long-term prognosis, serving as an extremely valuable tool for clinical management.

A previous study by Razmjoee et al. showed that in patients undergoing coronary artery bypass grafting, continuing care for two months was associated with a higher quality of life (QOL) and physical and mental dimensions scores than patients who received routine postoperative management.^10^ A recently published report indicated that implementing the continuing care model significantly increased the QOL scores of HF patients.^28^ The continuing care program improves quality of life in CHD patients by addressing multiple factors such as hospitalization frequency, diet, activities, medications, and risk factors.^11^ Moreover, in patients with chronic HF, continuing care for three months dramatically improves physical and mental quality of life compared to usual care.^29^ In general as per with these reports, this study reported that the continuing care led to a marked improvement in the HPLP-II and SF-36^30^-^32^ in patients with CHD combined with HF after PCI, further confirming a marked improvement in the QOL that is associated with implementing this model of care.

Furthermore, continuing care for three months was shown to improve cardiac function of HF patients, as manifested by increased LVEF and exercise capacity yet declined NT-proBNP levels.^29^ A significant difference was observed between the intervention and control groups in lung cancer patients with CHD post-PCI (continuing care and routine care, respectively) in terms of oxygen uptake, LVEF, 6-MWT, and medication compliance.^15^ In agreement with the previous results, this study showed that the continuing care program was associated with the increased LVEF and 6-MWT and diminished NT-proBNP levels compared to the routine postoperative care. Together, these results confirm that the continuing care program can effectively improve cardiac function in patients with cardiovascular diseases. The beneficial effects observed in the continuing care group may be attributed to multiple synergistic mechanisms. Firstly, improved medication adherence—reinforced through regular contact, digital reminders, and community-based follow-up—likely enhanced long-term hemodynamic stability and reduced recurrent ischemic events.^28^,^29^ Secondly, lifestyle modifications promoted by continuing care, including diet optimization, increased physical activity, and smoking cessation, contribute to attenuated neurohormonal activation and improved vascular endothelial function.^30^ Lastly, timely recognition and response to symptom changes may have facilitated early intervention, thereby preventing disease progression and promoting reverse cardiac remodeling.^31^,^32^ These mechanisms may collectively account for the reduced incidence of MACEs and improvements in cardiac parameters in LVEF and NT-proBNP. These results also resonate with emerging evidence from multicenter studies indicating that digital tools—such as app-based reminders, WeChat-managed follow-ups, and wearable devices—can effectively improve medication adherence and reduce post-discharge complications in cardiovascular patients.^13^,^14^ The adoption of digital strategies in our care model (e.g., monthly WeChat check-ins and educational video sharing) may have contributed to the improved outcomes observed in this study.

Left ventricular remodeling is a frequent event occurring in patients suffering from cardiovascular diseases after receiving PCI.^33^,^34^ In this study, all participants were followed up six months after PCI to record ventricular remodeling, as indicated by the LVEDV ≥ 20% increase from baseline six months after PCI.^35^ The incidence rates of ventricular remodeling were markedly lower in the continuing care group, 17.20% versus 30.00% in the routine care group. This result further confirms that the continuing care model can effectively reduce the incidence of ventricular remodeling in patients with CHD complicated by HF who are undergoing PCI.

This study also revealed the potential of continuing care to decrease the incidence of MACEs, as evidenced by lower incidence rates of MACEs in the continuing care group than in the routine care group (44.09% versus 66.67%). MACE is defined as a composite of unstable angina, stroke, myocardial infarction, late revascularization, and all-cause mortality.^36^,^37^ Recently published research shows that, despite timely treatment, patients with acute ST-segment elevation myocardial infarction undergoing primary PCI are at an increased risk of MACEs.^38^ MACEs are also highly prevalent in patients with acute coronary syndrome receiving PCI.^39^,^40^ This study confirms that continuing care is highly effective in preventing the development of MACEs in CHD patients with HF undergoing PCI.

This study offers additional insights into the potential benefits of continuing care for patients with CHD combined with HF after PCI. While several studies have addressed aspects of post-PCI management, few have concurrently examined both subjective outcomes (such as quality of life) and objective indicators (including LVEF, NT-proBNP, and MACEs) within a structured nursing care framework in a real-world setting. Our findings may help enrich the growing body of literature supporting integrated, patient-centered post-discharge care. From a clinical perspective, the continuing care model appears to be a feasible and cost-effective strategy to enhance treatment adherence, support cardiac recovery, and reduce long-term cardiovascular risk. The strengths of this study include well-defined inclusion criteria, a relatively consistent six month follow-up period, and a comprehensive assessment of clinical and functional outcomes. Nonetheless, limitations such as its retrospective design and single-center scope may restrict generalizability. Future research should involve multicenter, prospective studies incorporating validated tools for adherence and psychological assessment, as well as digital monitoring platforms, to further verify and extend these findings.

### Limitations:

First, it was a single-center retrospective analysis, and although the sample size was sufficient for the primary outcome comparisons, the generalizability of the findings remains limited. Second, psychological assessments such as PHQ-9 or GAD-7 were not included, as these were not routinely collected during the study period. Consequently, our evaluation of quality of life primarily focused on physical and behavioral dimensions. We acknowledge this limitation and plan to incorporate validated psychological instruments in future prospective studies to enable a more comprehensive assessment of patient well-being. Third, we did not report the domain-specific subscale scores of the SF-36 in order to maintain statistical clarity and minimize the risk of Type-I error associated with multiple comparisons. Future studies may benefit from more detailed analysis at the domain level to better identify which health dimensions are most affected by the intervention. Fourth, no sample size estimation or power analysis was conducted. Given the retrospective nature of the study, the sample size was determined by the available number of eligible patients. We chose not to perform a post hoc power calculation to avoid potentially misleading interpretations. Nevertheless, we plan to incorporate formal power analysis into future prospective designs to strengthen methodological rigor. Finally, the study did not include standardized adherence assessment tools such as the MMAS-8, as structured adherence data were not systematically collected in this retrospective analysis. However, the improvements observed in MACEs and LVEF may indirectly suggest enhanced adherence in the intervention group. Future prospective studies should incorporate validated adherence measures to better elucidate behavioral mechanisms underlying the observed benefits.

## CONCLUSION

The current research elucidates that in patients with CHD complicated by HF undergoing PCI, continuing care is efficacious in improving QOL and cardiac function. Moreover, continuing care can reduce the incidence of ventricular remodeling and MACEs. Continuing care, therefore, may have important implications for the clinical management and prognosis of CHD combined with HF.

### Authors’ contributions:

**CH:** Study design and manuscript writing, revision, validation and is responsible for the integrity of the study.

**DL and JL:** Data collection, data analysis and interpretation. Critical review.

All authors have read and approved the final manuscript.
